# The role of molecular tumor boards in neuro-oncology: a nationwide survey

**DOI:** 10.1186/s12885-024-11858-x

**Published:** 2024-01-19

**Authors:** Lisa S. Hönikl, Sebastian Lange, Vicki M. Butenschoen, Claire Delbridge, Bernhard Meyer, Stephanie E. Combs, Anna Lena Illert, Friederike Schmidt-Graf

**Affiliations:** 1 Department of Neurosurgery, Klinikum rechts der Isar, Technical University of Munich (TUM), Ismaninger Str. 22, 81675 Munich, Germany; 2 Center for Personalized Medicine (ZPM), Klinikum rechts der Isar, Technical Universitiy of Munich (TUM), Munich, Germany; 3 Department of Medicine II, Klinikum rechts der Isar, Technical University of Munich (TUM), Munich, Germany; 4 Department of Neuropathology, Institute of Pathology, Technical University of Munich (TUM), Munich, Germany; 5 Department of Radiation Oncology, Klinikum rechts der Isar, Technical University of Munich (TUM), Munich, Germany; 6 Department of Medicine III, Faculty of Medicine, Klinikum Rechts der Isar, Technical University Munich (TUM), Munich, Germany; 7 Department of Neurology, Klinikum rechts der Isar, Technical University of Munich (TUM), Munich, Germany

**Keywords:** Neuro-oncology, Molecular tumor board, MTB, Target therapy, Personalized medicine, Precision oncology

## Abstract

**Background:**

In neuro-oncology, the inclusion of tumor patients in the molecular tumor board has only become increasingly widespread in recent years, but so far there are no standards for indication, procedure, evaluation, therapy recommendations and therapy implementation of neuro-oncological patients. The present work examines the current handling of neuro-oncological patients included in molecular tumor boards in Germany.

**Methods:**

We created an online based survey with questions covering the handling of neuro-oncologic patient inclusion, annotation of genetic analyses, management of target therapies and the general role of molecular tumor boards in neuro-oncology in Germany. We contacted all members of the Neuro-Oncology working group (NOA) of the German Cancer Society (DKG) by e-mail.

**Results:**

38 responses were collected. The majority of those who responded were specialists in neurosurgery or neurology with more than 10 years of professional experience working at a university hospital. Molecular tumor boards (MTB) regularly take place once a week and all treatment disciplines of neuro-oncology patients take part. The inclusions to the MTB are according to distinct tumors and predominantly in case of tumor recurrence. An independently MTB member mostly create the recommendations, which are regularly implemented in the tumor treatment. Recommendations are given for alteration classes 4 and 5. Problems exist mostly within the cost takeover of experimental therapies. The experimental therapies are mostly given in the department of medical oncology.

**Conclusions:**

Molecular tumor boards for neuro-oncological patients, by now, are not standardized in Germany. Similarities exists for patient inclusion and interpretation of molecular alterations; the time point of inclusion and implementation during the patient treatment differ between the various hospitals. Further studies for standardization and harmonisation are needed. In summary, most of the interviewees envision great opportunities and possibilities for molecular-based neuro-oncological therapy in the future.

**Supplementary Information:**

The online version contains supplementary material available at 10.1186/s12885-024-11858-x.

## Background

Tumor boards/multidisciplinary cancer meetings have been established in neuro-oncology for a long time. Not least due to the increasing spread of certifications for neuro-oncological tumor centers in Germany, regular meetings to discuss neuro-oncological patients, implementation and documentation of interdisciplinary tumor boards according to standards are specified. There have also been international standards and guidelines for the implementation of tumor board conferences in neuro-oncology [1], combined with guidelines for diagnostics and therapy in neuro-oncology. The fact that molecular markers play an important role in neuro-oncology has not only been known since the last WHO classification in 2021 [2]. Rare innovative clinical trials like the NOA-20 study investigate molecular-matched therapeutic strategies in neuro-oncological tumors (glioblastoma) with an umbrella trial concept [3]. The best-known examples of relevant markers in neuro-oncology are IDH1/2 mutations and presence of LOH 1p/19q, which play an indispensable role in diagnostics and prognosis as well as therapy [4]. Much more is known at the molecular level in neuro-oncology and is constantly being further developed. This is associated with the hope of finding and establishing more targeted therapies. So far, many of these molecular properties in neuro-oncological patients have only been determined within the framework of molecular tumor boards or studies.

Molecular tumor boards (MTB) have been established in most academic centers in the last years. Molecular tumor boards are multidisciplinary teams consisting of medical professionals, including oncologists, geneticists, pathologists, and other specialists. They convene to discuss and analyze the genetic and molecular makeup of a patient’s tumor. These boards utilize advanced technologies to scrutinize the genomic data of the tumor, aiming to tailor personalized treatment strategies based on the specific genetic alterations present in the cancer. The primary goal of molecular tumor boards is to determine the most effective and precise therapeutic approaches, considering the individual’s unique genetic profile for better outcomes in cancer treatment. In the case of certain tumor entities (e.g. non-small cell lung cancer, soft tissue sarcoma), patients are already regularly included and, in some cases, successfully treated with targeted substances. In neuro-oncology, the inclusion of tumor patients in the MTB has only become more widespread in recent years [1, 5–8]. But so far, there are not enough standards for indication, procedure, evaluation of molecular findings, therapy recommendations and therapy implementation. The first EANO guideline for molecular testing in glioma for targeted therapy selection has just been published [9]. The implementations of MTB and targeted therapy are made more difficult, not least because of the lack of standards and evidence, by the often unwillingness of health insurance companies to assume the costs of molecular analysis.

We conducted a survey on molecular tumor boards in neuro-oncology to capture current practice at multiple neuro-oncology centers in Germany. Potential issues with inclusion and implementation, as well as future requirements and needs should be identified and highlighted in order to work on improving and standardizing molecular tumor boards in neuro-oncology.

## Methods

### Study design

A questionnaire with 25 questions was created. The first five questions addressed the professional experience and speciality in which the respondents worked, as well as the availability of including neuro-oncological patients to the MTB. Seven questions followed about the basic handling of the MTB and the infrastructure of the respective hospitals. Afterwards the annotation criteria, possible application of therapy and cost takeover were asked with further 11 questions. At the end of the survey, a personal assessment of the MTB could be given in two open questions. The survey consisted of 16 single-choice, seven multiple-choice and two open questions.

### Questionnaire

Members of the departments of neurosurgery and neurology and those responsible for the neuro-oncological part of MTB created the questionnaire. It was reviewed by all authors and approved by the board members of the Neuro-Oncology working group (NOA) for understandability and significance. An ethical vote was not necessary, as there were no patients or clinical data included.

The survey was accessible as an online-based questionnaire at www.survio.com. Invitations for the survey were sent by e-mail to all 445 members of the Neuro-Oncology working group (NOA, “Neuroonkologische Arbeitsgruppe”) of the German Cancer Society (DKG, “Deutsche Krebsgesellschaft”). The participation was possible from 4th October 2022 to 31st October 2022 (4 weeks).

## Results

### Responses and participants background

**Fig. 1:**
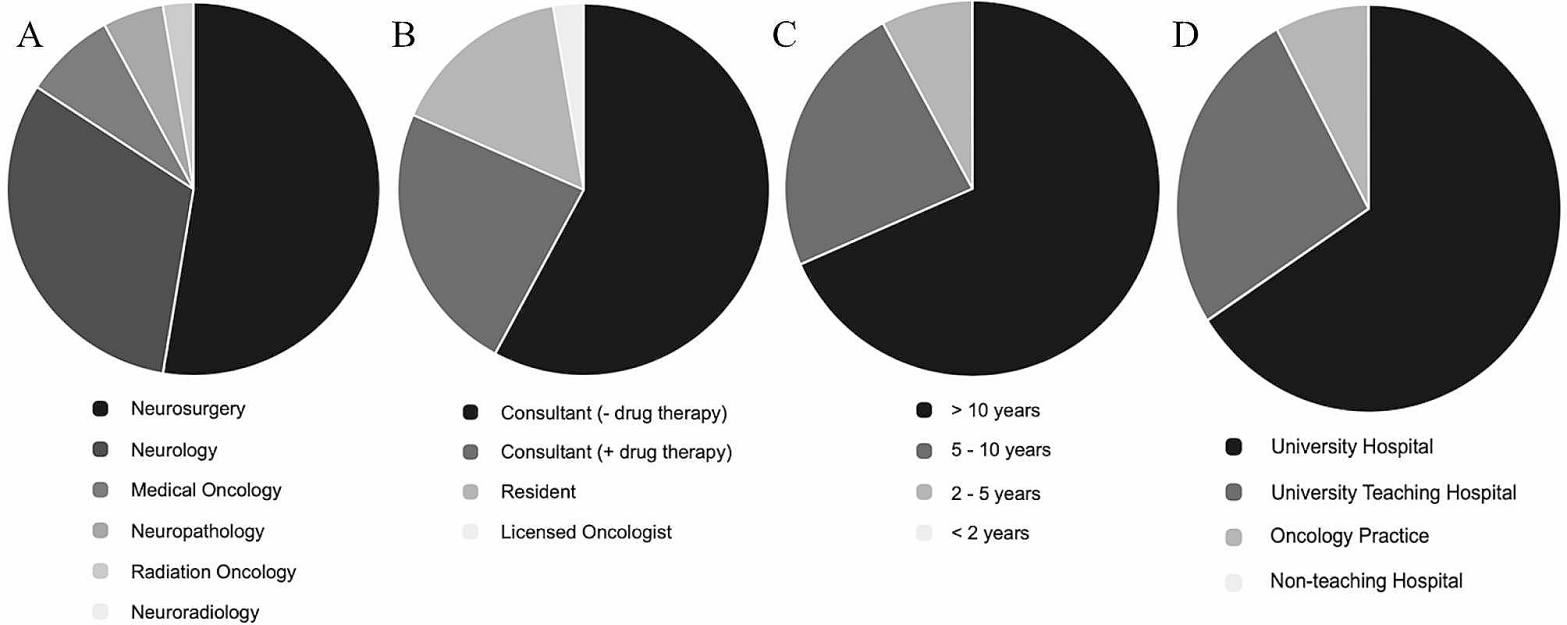
Composition of the participating cohort. Responders were mostly neurosurgeons or neurologists (**A**) in supervising positions (**B**) with more than 10 years of professional experience (**C**) and working in University hospitals or University teaching hospitals (**D**)

We counted 115 visits and 38 completed surveys (8.5%) of 445 possible participants. The majority of responders were neurosurgeons (20/38, 52.6%) followed by neurologists (12/38, 31.6%), medical oncologists (3/38, 7.9%), neuropathologists (2/38, 5.3%) and radiation oncologists (1/38, 2.6%) (Fig. 1A).

Most of them were specialists (31/38, 81,6%), 9 of them (23,7%) with additional qualification in drug-based tumor therapy. Furthermore, 6 residents (15.8%) and one licensed neuro-oncologist in an ambulant practice (2.6%) answered the survey (Fig. 1B).

Over 90% of the participants have more than 5 years professional experience (> 10 years 26/38, 68.4%, 5–10 years 9/38, 23.7%) (Fig. 1C).

The majority of interviewees work at university hospitals (25/38, 65.8%) followed by university teaching hospitals (10/38, 26.3%) and oncology practice or medical care center (3/38, 7.9%) (Fig. 1D). None of the participants worked in a non-teaching hospital. Therefore, many of these are part of a certified neuro-oncology center (27/38, 71.1%).

### Molecular tumor boards in Germany

More than half of the participants have a molecular tumor board directly at the hospital they work (25/38, 65.8%). 34.2% have access to the MTB as referring physicians (13/38). Mostly, the MTB take place once a week (weekly 25/38, 65.8%; every two weeks 5/38, 13.2%; once a month 8/38, 21.1%).

In most of the molecular tumor boards at the various hospitals, according to this survey, the multidisciplinary treatment team of a neuro-oncology patient is represented in the meetings with colleagues from neuropathology (27/38, 71.1%), neurology (26/38, 68,4%), neurosurgery (21/38, 55.3%), radiation oncology (19/38, 50.0%), medical oncology (26/38, 68.4%), neuroradiology (13/38, 34.2%), as well as the person who reports the diagnostic findings and the molecular tumor board recommendation (22/38, 57.9%).

50.0% stated that the MTB recommendations are presented and discussed in the (already existing) neuro-oncology tumor board (19/38), whereas 44.7% of the responders quote that the results are discussed in a separate entity independent molecular tumor board (17/38). 2 responders state that their recommendations are discussed in an entity dependent neuro-oncological molecular tumor board (2/38, 5.3%).

The NCT/DKFZ/DKTK MASTER (Molecularly Aided Stratification for Tumor Eradication) Program is a nationwide program for multidimensional characterization of patients with advanced rare cancers where targeted tumor therapies are recommended based on next generation sequencing [10, 11]. Neuro-oncology patients are not regularly included in the Germany-wide “Master Program” (never: 20/38, 52.6%; 1–2 patients a year: 8/38, 21.1%; 3–4 patients a year: 1/38, 2.6%; >4 times a year: 9/38, 23.7%).

31,6% (12/38) regularly include neuro-oncological patients in the MTB with a defined indication (e.g. diagnosis, age, lack of treatment option). 23,7% (9/38) include all patients based on the definition of a specific entity. Regularly (repeated times with similar diagnosis/ situation), but only if there is no further therapy option, patients are included by 18,4% (7/38) of the respondents. 21,1% (8/38) rarely (not often/ seldom) refer patients to the MTB.

**Fig. 2:**
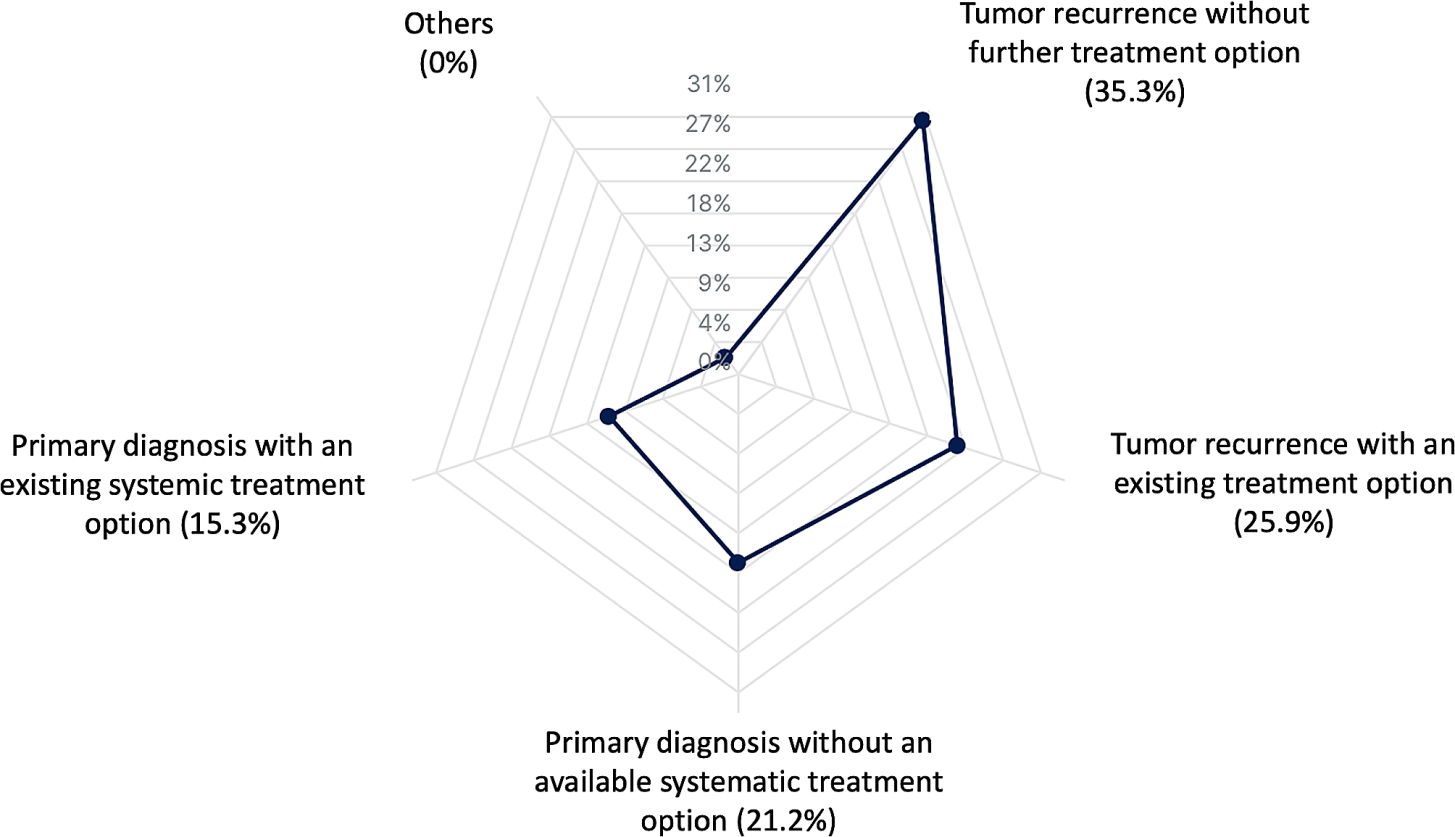
Overview of inclusion criteria for the MTB

A clearly defined inclusion criteria for MTB does not exist in Germany. Mostly, neuro-oncological patients with tumor recurrence were included (Fig. 2): recurrence without any standard therapy in 78.9% (30/38), recurrence with further therapy options in 57.9% (22/38) of the respondents. Patients with initial tumor diagnosis without any standard therapy regimen were included in 47.4% to the MTB (18/38). In 34.2% (13/38), patients with neuro-oncological diseases with standard therapy regimen are included at the time of initial diagnosis.

The most common diagnosis of patients in MTB are glioblastoma, diffuse midline glioma, astrocytoma WHO CNS grade 4 and 3 (Table 1). In Table 1 there is an overview of the tumor entities which are included in the molecular tumor board according to the responders.

**Table 1:** Overview of neuro-oncology tumor entities which are included in the molecular tumor board

Diagnosis	Response	Percentage
Glioblastoma WHO CNS grade 4	30/38	78,9%
Diffuse midline glioma WHO CNS grade 4	28/38	73,7%
Astrocytoma WHO CNS grade 4	26/38	68,4%
Astrocytoma WHO CNS grade 3	23/38	60,5%
Astrocytoma WHO CNS grade 2	16/38	42,1%
Oligodendroglioma WHO CNS grade 3	21/38	55,3%
Oligodendroglioma WHO CNS grade 2	15/38	39,5%
Ependymoma WHO CNS grade 3	21/38	55,3%
Ependymoma WHO CNS grade 2	14/38	36,8%
Meningioma WHO CNS grade 3	21/38	55,3%
Meningioma WHO CNS grade 2	14/38	36,8%
Chordoma	16/38	42,1%
Others	07/38	18.4%

### Variant interpretation, recommendation and implementation in tumor therapy

The variant interpretation and creation of recommendations are done by members of the MTB with another speciality (different than the neuro-oncological attending physicians) (17/38, 44.7%), neurosurgeons (14/38, 36.8%), neurologists (9/38, 23.7%), oncologists (9/38, 23.7%), member of the MTB with only responsibility for the annotation of the cases without clinical care of the patients discussed (6/38, 15.8%). In 28,9% (11/38), the first annotations and interpretations are done by a specialist or senior physician.

The frequency of given MTB recommendations is very heterogeneous within this survey: 34,2% give recommendations for 80–100% of the MTB cases, 34,2% give recommendations for 50–80% and 31,6% give recommendations for less than 50% of MTB patients.

47.4% state that they implement the therapeutic MTB recommendations in 80–100% (18/38), followed by 23.7% who point out that they implement the therapeutic MTB recommendations in less than 30% (9/38), 18,4% (7/38) for 50–80% implementation of therapeutic MTB recommendations, 10,5% (4/38) for 30–50% implementation.

Asking how many patients already died before the recommended therapy could be implemented, only 2,6% (1/38) stated that this had happened in 80–100% of cases. In 13,2% (5/38), this had happened in 50–80% of the patients, in 21,1% (8/38), 30–50% of the patients had died, and in 63,2% (24/38), this had happened in < 30% of the cases.

The cost takeover for the molecular analyses within the MTB is regularly no problem for most of the respondents (no problem: 13/38 (34.2%), rarely a problem 12/38 (31.6%)). For all others, there are more problems with cost coverage for patients with general health insurance (28,9%) than with a private insurance (5,3%). Cost coverage for experimental therapies on an off-label single case basis is quite more difficult: many of the interviewees report that it is difficult to get a cost coverage for experimental therapies independently the insurance status (16/38, 42.1%), whereas 28.9% (11/38) have rarely, 13.2% (5/38) have no and 15.8% (6/38) only problems with general health insured patients.

Most of the responders of our survey do not know which evidence level of therapy studies/ literature is necessary for a targeted therapy recommendation at their molecular tumor board (14/38, 36.8%). Pure preclinical studies (1/38, 2.6%) or experimental associations between molecular pathways (1/38, 2.6%) seemed not sufficient for treatment recommendation for most of the interviewees. The responses for all other evidence levels are very heterogenous: prospective study in the same entity (4/38, 10.5%), retrospective study in the same entity (5/38, 13.2%), case report in the same tumor entity (3/38, 7.9%), prospective study in another tumor entity (3/38, 7.9%), retrospective study in another tumor entity (4/38, 10.5%) or case report in another tumor entity (3/38, 7.9%). In summary, a possible cut off for a therapy recommendation is a retrospective study in another tumor entity (median 9.2%).

MTB recommendations are regularly given for alteration classes: pathogenic alteration (class 5: 17/38, 44.7%) and likely pathogenic alteration (class 4: 15/38, 39.5%). 36.8% of the interviewees do not know which alteration class is necessary in their molecular tumor board. Only few responded that they also give recommendations for benign alterations (class 1: 1/38, 2.6%) or variants with unknown significance (class 3: 2/38, 5.3%).

Regarding the implementation of therapies, most experimental therapies are given at the department of medical oncology (27/38, 71.1%), followed by neurology (15/38, 39.5%), neurosurgery (10/38, 26.3%) and radiation oncology (10/38, 26.3%) (multiple answers possible).

Experimental therapies implemented as part of neuro-oncology MTB treatment were considered by respondents to be superior (9/38, 23.7%) or equivalent (9/38, 23.7%) to therapies used in the past. 13.2% state that the therapy was less successful than the therapy performed before (5/38). In 10.5% (4/38), no experimental therapy was performed so far or not well tolerated in 7.9% (3/38). 21.1% have “other experience” than the mentioned (8/38).

### Outlook and current problems

Some of the most frequently mentioned points from the open questions are summarized below. Predominantly, the future of the MTB is seen positively. A lot of the responders mentioned that the MTB is expandable. Several participants wrote that molecular tumor boards are the future for neuro-oncology patients.

Most frequently mentioned points: There should be more sequencing, more implementation and awareness for the option of targeted therapies. Problems with cost takeover (for MTB and/ or treatment) should be more discussed. Patients should be included to the MTB earlier within their course of disease. More experimental medication studies are needed. A standardisation within Germany is necessary.

When asked about the future of the molecular tumor board for neuro-oncological patients, almost all respondents were optimistic. MTB is seen by most as an integral future part of therapy planning, but significant improvements and advances are also needed. With an increasing number of new markers and targets, new therapeutic options are expected. In particular, young patients in good condition should be given the opportunity to be included early in their course of disease. For all patients, early inclusion should be possible, also in order to combine new therapies with standard procedures. There should be clear inclusion criteria for neuro-oncological patients, and access to MTB should be facilitated. The assumption of costs for MTB and experimental therapies by health insurance companies should also be based on clear criteria and standards. Regular evaluations of the results of the MTB and therapies used are required and necessary. Only two respondents see MTB in neuro-oncology rather negatively and only find it as useful for a very small proportion of patients.

In further comments, it was most frequently stated that the assumption of costs by the health insurance companies should be made easier in order to facilitate and speed up access to the MTB and the entire procedure. Great importance is attached to standards in the indication but also in the evaluation with annotation and therapy recommendations for the MTBs. Furthermore, the desire for early inclusion of neuro-oncological patients in the MTB was emphasized. In addition, many patients should be included in order to gain experience. In the evaluation and recommendation, information about ongoing studies should be provided; umbrella and basket studies in particular are seen as necessary.

## Discussion

### Survey participants

We conducted a multi-center and multi-disciplinary survey about molecular tumor boards (MTB) in Germany by sending an e-mail with a link to all members of the Neuro-oncology working group (NOA) of the German Cancer Society (DKG). Overall, only a relatively small number of NOA members answered the questionnaire, which is consistent with the fact that only a very limited number of neuro-oncological physicians are involved in molecular tumor boards or are familiar with them at all. Due to the low number of participants in the survey, the results must be viewed cautiously and only provide a rough indication of the current status.

There are 53 certified centers for neuro-oncology in Germany (according to the DKG homepage), which do not necessarily have to be connected to a molecular tumor board. With 36 medical universities in Germany (Source: Federal Statistical Office 2020) and 15 Comprehensive Cancer Center of Excellence (CCC) it is to be expected that a molecular tumor board will exist across all entities at each CCC. Certification of Molecular Tumor Boards in the context of the establishment of Centers for Personalized Medicine is possible since the end of 2022 by the DKG. To date (05/2023), only 3 university hospitals in Germany are certified centers for personalized medicine (“ZPM”; Charité Berlin, University Hospital Freiburg and TU Munich). Most university centers have a certified neuro-oncological center. It can be assumed that there are only 1–3 colleagues with neuro-oncological knowledge and expertise in the molecular tumor board, which roughly corresponds to the number of specialists who took part in the survey.

So, one main limitation of our survey could be, that the results are probably not fully representative for all molecular tumor boards in Germany because of the low response rate. However, e-mail-communications by several of the responders suggest that in the majority of centers, only one (responsible) person per center responded to the survey.

The majority of physicians participating in the study are neurosurgeons and neurologists, presumably the colleagues who treat and provide follow-up care for the neuro-oncological patients, and mostly the colleagues who register the patients in the molecular tumor board, but also coordinate the results and further procedures, if possible and/ or necessary.

Most of the participants are specialists, which also often includes more than 10 years of experience in neuro-oncology. Most of the participating physicians work at the university hospital, and an even larger proportion work in a certified neuro-oncological center. Almost 66% have access to the molecular tumor board in the center where they work, all others as referrers.

### Process of MTB and infrastructure around

The molecular tumor board normally takes place once a week, but the increasing demand already seems to determine the regular and frequent meetings. Participation is stated to be interdisciplinary, as expected, primarily with neuropathologists, oncologists, neurologists followed by neurosurgeons and radiotherapists and neuroradiologists.

In half of the participants, molecular findings of the MTB are discussed as part of the neuro-oncological tumor board, at the other centers this is usually done within an entity-independent MTB, which in both cases at least contributes to the fact that a larger and interdisciplinary team is involved. It is also shown that input about neuro-oncological procedures, but also the clinical situation of individual patients, as well as general clinical know-how, are important facts, but also oncological and neuropathological knowledge is useful and absolutely necessary. The fact that one third of the respondents did not have an internal oncologist present at the MTB should be avoided by appropriate regulations and interdisciplinary cooperation should be further intensified. The possible advantages and disadvantages of integration into a general-interdisciplinary tumor board versus a specific neuro-oncological molecular tumor board are summarized in Table 2.

**Table 2:** Advantages and disadvantages of general MTBs vs. specialized neurooncological MTBs

general-multidisciplinary TB		specialized neurooncological-MTB	
Advantage	Disadvantage	Advantage	Disadvantage
Greater and multiprofessional Team/know how	Less know-how about targeted therapies in neurooncological cases	Great know-how about neuro-oncological diseases and actual therapy studies	Missing experience regarding the effectiveness/application of specific and targeted medications in other entities.
Greater presence of targeted therapies for everyone	Less time for detailed discussion regarding targeted therapies	Few cases per session, hence more time for discussion	During a separate meeting, the presence in the interdisciplinary treatment team of the patient is missing.

Although it has been shown here that physicians with neuro-oncology experience participate in molecular tumor boards -which discusses neuro-oncological patients- and that access to these boards is easy for all treating physicians, clear guidelines for participation with at least one reporting physician, one neuropathologist/ pathologist, and one clinically experienced neuro-oncologist are elementally helpful and are thus mandatory for certification of centers for personalized medicine (ZPM). Renovanz et al. published [12] their data of 408 patients with neuro-oncological tumors presented at the MTB. They stated the MTB as a linchpin, consisting of clinicians from all oncology disciplines, pathologists, neuropathologists, pharmacologists, cancer biologists, geneticists and bioinformatics experts with a comprehensive workflow on the clinical course and outcome of patients. Many of the necessary and demanding standards of MTB and the structures around can be found here.

About half of those surveyed (47.4%) include at least a few patients in the Germany-wide master program. The NCT/DKFZ/DKTK MASTER (Molecularly Aided Stratification for Tumor Eradication) Program is a nationwide program for multidimensional characterization of patients with advanced rare cancers where targeted tumor therapies are recommended based on next generation sequencing [11].

Only about one third of patients are included in a MTB according to clear guidelines, which shows the uncertainty about indication and standards in molecular tumor boards in the neuro-oncology field. In some cases, individual patient groups are regularly included according to specific criteria (31.6%), and in 18.4% patients are also regularly included due to lack of further treatment options. Overall, higher WHO classifications are observed to lead to inclusion more frequently (see Table 1). This heterogeneity demonstrates the importance of studies and surveys on molecular analyses and therapies to generate better and more uniform standards. Within the funded project “German Network Personalized Medicine” (DNPM) [13] as well as the DKTK MTB-Alliance a harmonization and standardization of MTB procedures, inclusion criteria and decisions is intended and defined.

It would also be desirable to develop specific inclusion criteria for neuro-oncologic patients and to raise molecular testing, if necessary, already in the early history, as well as in patients with a lower WHO classification, in whom valuable and novel treatment options could be discovered and considered at an early stage. Conversely, neuro-oncologic entities and constellations in which molecular testing is not promising should be identified by consistent data collection to save resources and costs and be more appropriately used in other patients. A frequent problem is also that patients have already died before possible implementation of therapy recommendations from the molecular tumor board, which is why entity-specific inclusion time windows should be developed.

Further reasons for the restrained inclusion of neuro-oncology patients in the molecular tumor board may include the limited number of targetable alterations. To date, there are too few clinical studies for the use of targeted therapy in neuro-oncological diseases. Additional studies for the implementation of targeted therapies are urgently needed. If this leads to further treatment options, the molecular tumor board would significantly enhance its importance in the treatment of neuro-oncological tumors.

### Annotation criteria, therapy recommendations, cost takeover

The information about the one who is doing the report with the molecular findings appears to be very diverse, in the range from those responsible without clinical activity to colleagues from other disciplines to the various neuro-oncological clinicians. Last but not least, this certainly has something to do with the time required to create reports, know-how and resources. In order to improve quality and comparability, it would also be desirable to have fixed specifications for reporting within the setting of molecular tumor boards.

The basis of targeted therapy recommendations is several clinical or preclinical evidence levels as well as the classification how pathogenic a genetic alteration is. Nearly 50% of the responders do not know the cut offs for sufficient good evidence for the MTB recommendations. We think that this also shows that we should have more molecular expertise to deal with the processes and results of the MTB in a responsible and meaningful way. Overall, the responses were very heterogeneous which shows up that there is no standardization by now.

In an astonishingly high proportion of patients, recommendations for therapy are made, most of which are implemented in over 50% of cases. Regular evaluation of the cases is all the more desirable and sensible. Initial retrospective evaluations of molecular tumor boards revealed significantly lower levels of implemented therapies [14, 15] (17% and 20%, for example). Reasons for no therapies were no mutations identified, no actionable mutations and clinical deterioration [15]. The prospective recording of performed therapies as well as the success of such therapies are essential for further evaluation and use of molecular tumor boards and targeted therapies in the future. The experimental therapies are mainly given in haemato-oncological departments (71.1%), which certainly have the greatest expertise in this area. Here, an interdisciplinary care of neuro-oncological patients should be supported and demanded.

Almost half of the survey participants state that targeted therapies are more successful or similarly effective than previously performed therapies for the respective entity, which underlines the corresponding importance of the molecular tumor board and corresponding recommendations. Nevertheless, it must be pointed out once again at this juncture that only a small fraction of the treating oncologists, neuro-oncologists, and neurosurgeons participated in the survey.

An important problem in Germany, as certainly in other countries [16], is the assumption of costs, both for molecular analysis and for the implementation of targeted therapies. At the time of inclusion in the molecular tumor board, this is a frequent problem in almost 30%, primarily in patients with statutory health insurance. More than 50% often have problems with the assumption of costs for experimental therapies after recommendation of the molecular tumor board, both in statutory and privately insured patients (42.1%) or, again, above all in patients with statutory health insurance (15.8%). There are not only laborious bureaucratic obstacles to overcome, but valuable time of the patient’s life is often lost and therapies have to be postponed or are no longer possible at all due to a deterioration in condition. Despite a globally recognized German healthcare system, there is a clear advantage for patients in private health insurance to get access to MTB and experimental therapies. In order to be able to establish and expand molecular examinations and therapies in the future for all patients, one of the most important points is the regulation of the assumption of costs and this according to clear criteria.

### Future perspectives

Overall, most participants are optimistic about the molecular tumor board. A further expansion of neuro-oncological cases and their analyzes is required, but also an improvement of the entire procedure. Fixed criteria for inclusion, indications, evaluation and recommendations as well as assumption of costs are required, as is interdisciplinary work on the board and in treatment. Not only clear criteria would be conducive to this, but also, for example, the basic possibility of online participation, cross-center exchange and evaluation of recommendations (e.g. comprehensive cancer centers/ CCC or bavarian cancer centers/ “BZKF”) as well as cross-center studies and projects on the use of therapy (e.g. bavarian cancer centers “BZKF”: collecting nonV600-BRAFmutated tumors). All this appears to be a prerequisite for further development of new markers and new targeted therapies.

An ideal neuro-oncology tumor board from our perspective includes a multidisciplinary team comprising neurosurgeons, neurologists, radiation oncologists, and oncologists. It should be highly present and regularly provide updates on the current state of research regarding potential targeted therapies for attending physicians. Ideally, through further studies, a greater number of potential treatment options can be discovered and implemented, allowing these therapies to become a part of the standard care for patients and easily applicable.

## Conclusion

Due to the expected positive further development of molecular markers and new experimental targeted therapies as well as the associated opportunities, standards for the inclusion, indication, investigation and interpretation of molecular alterations, therapy recommendations and therapy implementation and implementation of molecular boards must be demanded from a clinical and scientific point of view. EANO guidelines on rational molecular testing of gliomas are the first step in the right direction [9]. Standardization and certification of molecular tumor boards currently focuses on structural and entity-specific aspects and has just started in Germany [14]. In order to achieve the greatest possible benefit for individual, difficult-to-treat patient cohorts, these standardized procedures for admission, indication, analysis and interpretation of molecular changes, therapy recommendations and implementation of molecular tumor boards should be adapted to the neuro-oncological patient´s cohort and importantly coordinated and rolled out with the corresponding neuro-oncological treatment leaders and teams.

Regulated assumption of costs for the implementation are not only a prerequisite for everything else, but also only possible through urgently needed standards. In summary, a great and important future for molecular based therapy in neuro-oncology is seen.

### Electronic supplementary material

Below is the link to the electronic supplementary material.

**Supplementary Material 1**: Survey about neuro-oncological molecular tumor boards

## Data Availability

All underlying data can be obtained from the corresponding author upon request.
