# Supplementary material for: The role of molecular tumor boards in neuro-oncology: a nationwide survey
Source: BMC Cancer. 2024 Jan 19;24:108. doi: 10.1186/s12885-024-11858-x (PMC10797778; doi:10.1186/s12885-024-11858-x)
Supplement: Supplementary file 1 — Supplementary Material 1: Survey about neuro-oncological molecular tumor boards [file 12885_2024_11858_MOESM1_ESM.docx]

**Survey about neuro-oncological molecular tumor boards**

1) What field do you come from?

- Neurology
- Neurosurgery
- Department of Radiation
- Neuropathology
- Neuroradiology
- Oncology / internal Medicine

2) What professional position are you in?

- Consultant
- Consultant spezialized for drug tumor therapy
- Resident
- Physician in private practice

3) How long have you had experience in neuro-oncology

- >10 years
- 5-10 years
- 2-5 years
- <2 years

4) Where are you working?

- University hospital
- Teaching hospital
- other hospital
- Practice, medical supply centre

5) Do you work in a certified neurooncological center?

- Yes
- No

6) Do you have a molecular tumor board?

- Yes, directly (in the hospital I work in)
- Yes, indirectly as referrer
- No

7) How often does the molecular tumor board take place in your hospital/practice?

- weekly
- every two weeks
- once a month

8) Are neurooncological patients included in the molecular tumor board at your hospital/practice?

- Yes, regularly
- Yes, rarely
- No

9) Who regularly takes part in the molecular tumor board? (multiple answers)

- Reporter
- Neurosurgeon
- Neurologists
- Radiation therapists
- Neuropathologist
- Neuroradiologist
- Hematooncologist/Oncologist
- Others

10) Are the neurooncological molecular tumor board findings discussed separately or as part of an entity-independent molecular tumor board?

- joint discussion of all sequencing recommendations in one molecular tumor board
- as part of the neurooncological tumor board
- Entity-specific discussion of neurooncological patients in their own event (independent of the general neurooncological tumor board)

11) Do you regularly include neurooncological patients in the Germany-wide master's program?

- Yes, 1-2 cases/year
- Yes, 3-4 cases/year
- Yes, >4 cases/year
- No

12) Which neurooncological tumor entities are included in your molecular tumor board? (multiple answers)

- Glioblastoma WHO CNS Grade 4
- Diffuse midline glioma WHO CNS Grade 4
- Astrozytoma WHO CNS Grade 4
- Astrozytoma WHO CNS Grade 3
- Astrozytoma WHO CNS Grade 2
- Oligodendroglioma WHO CNS Grade 3
- Oligodendroglioma WHO CNS Grade 2
- Ependymoma WHO CNS Grade 3
- Ependymoma WHO CNS Grade 2
- Meningeoma WHO CNS Grade 3
- Meningeoma WHO CNS Grade 2
- Chordoma
- Others (free text)

13) In which therapy line are the patients registered for the molecular tumor board? (Multiple answers)

- Primary diagnosis with possible standard therapy
- Primary diagnosis without standard therapy
- Tumor recurrence with further therapy options
- Tumor recurrence without further therapy option
- Others (free text)

14) Who is responsible for the annotation of the cases? (multiple answers)

- A Person, who is only responsible for the annotation of the cases, without clinical work
- A doctor from another specialization
- Neurologist
- Neurosurgeon
- Consultant
- Resident
- Others (free text)

15) How often do you get recommendations?

- 80-100% of the cases
- 50-80% of the cases
- <50% of the cases

16) How often are recommendations implemented in the patient`s tumor therapy?

- 80-100% of the cases
- 50-80% of the cases
- 30-50% of the cases
- <30% of the cases

17) How often have your patients died before the MTB recommendations were implemented?

- 80-100% of the cases
- 50-80% of the cases
- 30-50% of the cases
- <30% of the cases

18) Do you regularly have problems with the assumption of costs for patient inclusion (payment of the molecular tumor board)?

- No
- Yes, especially for privately insured patients
- Yes, especially for patients with statutory health insurance

19) Do you regularly have problems with the assumption of costs for experimental therapies?

- No
- Yes, especially for privately insured patients
- Yes, especially for patients with statutory health insurance

20) At what level of evidence recommendation for a targeted therapy is made and prognostically implemented in the tumor therapy of your patient?

- M1A: in the same tumor entity, the predictive value of the biomarker or clinical efficacy in a biomarker-stratified cohort is indicated in an adequately powered prospective study or meta-analysis.
- M1B: in the same tumor entity, the predictive value of the biomarker or the clinical efficacy in a biomarker-stratified cohort in an adequately powered prospective study (marker secondary endpoint) or a retrospective cohort or case-control study is to be demonstrated.
- M1C: in the same tumor entity, in the presence of the biomarker, clinical efficacy has been demonstrated in one or more case reports.
- M2A: in other tumor entities predictive value of the biomarker or clinical efficacy in a biomarker-stratified cohort is indicated in an adequately powered prospective study or meta-analysis.
- M2B: in other tumor entities is the predictive value of the biomarker or clinical efficacy to be demonstrated in a biomarker-stratified cohort in an adequately powered prospective study (marker secondary endpoint) or in a retrospective cohort or case-control study.
- M2C: in other tumor entities, if the biomarker is present, clinical efficacy has been demonstrated in one or more case reports.
- M3: preclinical data ((in vitro/in vivo models), functional genetic studies) show an association of the biomarker with the efficacy of the medication, which is supported by a scientific rationale.
- M4: A scientific rationale suggests an association of the biomarker with the effectiveness of mediation, which has not yet been supported by (pre-)clinical data.
- I don't know the level of evidence which is used in my hospital

21) For which alteration classes do you regularly make recommendations? (multiple answers)

- Class 1: benign alteration
- Class 2: probably benign alteration
- Class 3: Variant of unclear significance
- Class 4: presumably pathogenic alteration
- Class 5: pathogenic alteration
- I don’t know the alteration class

22) Who implements recommended (experimental) therapies? (multiple answers)

- Hemato-/ Oncologist
- Neurologist
- Neurosurgeon
- Radiation therapist
- Others (free text)

23) What is your experience/impression (if no success evaluation is available) regarding the response of targeted therapies in neurooncology?

- Successful so far, OS > standard therapy
- Successful so far, OS = standard therapy
- Little success so far, OS < standard therapy
- Little success so far, mainly due to poor tolerability and low response
- No targeted therapy administered by now
- Others: (free text)

24) How do you see the future of neurooncological molecular tumor boards?

- (free text)

25) Do you have any further comments regarding the molecular tumor board for neuro-oncological patients?

- (free text)
